# Stochastic processes govern gut bacterial community assembly in a *Schistosoma mansoni*-transmitting snail, *Biomphalaria straminea*

**DOI:** 10.1371/journal.pntd.0012828

**Published:** 2025-02-05

**Authors:** Zhanhong Yuan, Jinni Hong, Jehangir Khan, Jinghuang Lu, Benjamin Sanogo, Zhongdao Wu, Xi Sun, Datao Lin

**Affiliations:** 1 Department of Parasitology, Zhongshan School of Medicine, Sun Yat-sen University, Guangzhou, China; 2 Key Laboratory of Tropical Disease Control, Ministry of Education, Guangzhou, China; 3 Provincial Engineering Technology Research Center for Diseases-vectors Control, Chinese Atomic Energy Agency Center of Excellence on Nuclear Technology Applications for Insect Control, Guangzhou, China; 4 Department of Traditional Chinese Medicine, Guangdong Provincial People’s Hospital, Guangdong Academy of Medical Sciences, Southern Medical University, Guangzhou, China; 5 Department of Zoology, Abdul Wali Khan University, Mardan, Pakistan; 6 Hainan General Hospital, Hainan Medical University, Haikou, China; 7 Laboratory of Parasitology, Institut National de Recherche en Sante Publique, Bamako, Mali; Oregon State University College of Veterinary Medicine, UNITED STATES OF AMERICA

## Abstract

**Background:**

Studies have revealed extensive taxonomic classifications and patterns of gut microbial diversity in snails, with limited focus on community assembly processes. To better understand the balance between stochastic and deterministic processes in the snail gut microbial assembly and their associations with snail fitness, we used the freshwater snail *Biomphalaria straminea* as a model and analyzed the gut bacterial communities from 118 samples via high-throughput sequencing of the 16S rRNA gene.

**Results:**

This study reveals that Proteobacteria and Bacteroidota dominate the gut microbiota of *B*. *straminea*. Snails from different laboratory habitats exhibit similar gut bacterial diversity but significantly different community structures. The assembly of gut bacterial communities in both laboratory and wild samples is predominantly influenced by stochastic processes rather than deterministic processes, as evidenced by the neutral community model (NCM). Furthermore, during the snail invasion and adaptation to a new environment, stochastic processes are more crucial than deterministic ones in shaping the snail gut microbiota. This indicates that the interplay between stochastic and deterministic processes in the snail gut microbial assembly is associated with host fitness during snail adaptation to a new environment. Based on the null model analysis, we also found that stochastic processes (based on dispersal limitation, homogenizing dispersal, and undominated processes) play a larger role than deterministic (based on homogeneous selection and variable selection) in driving the snail gut bacterial community assembly. Furthermore, the significant difference in the proportions of dispersal limitation and undominated processes is linked to both adaptive and non-adaptive snails.

**Conclusions:**

This study demonstrates that stochastic processes govern the assembly of the gut microbiota in *B*. *straminea*. Furthermore, snail adaptation is associated with the interplay between stochastic and deterministic processes in gut microbial composition. This study provides a better understanding of the dynamic patterns of the gut microbial community in freshwater gastropods and may contribute to the development of strategies for controlling intermediate hosts and schistosomiasis.

## Author summary

*Biomphalaria straminea*, an important intermediate host for the blood fluke *Schistosoma mansoni*, has invaded China since 1973. Here, our study explores the complex interplay of stochastic and deterministic processes in shaping gut bacterial communities within *B*. *straminea*. We identify dominant gut microbiota and reveal the pronounced influence of stochastic processes from 118 samples, particularly during snail invasion and environmental adaptation. This research contributes new insights into the dynamics of gut microbial communities in freshwater gastropods, emphasizing the ecological impacts on host adaptation. With implications for controlling intermediate hosts and mitigating schistosomiasis transmission, our findings hold the potential for addressing this neglected tropical disease.

## Introduction

Organisms, including vertebrates and invertebrates, harbor a diverse gut microbiota, often referred to as the host’s ‘second genome’ [1]. The advent of next-generation sequencing technology has greatly enhanced our understanding of the gut microbiota and human health [2–5]. In recent years, the gut microbial assembly of animals such as mice and fish has been established [6–12]. These studies have demonstrated that the gut microbiota may promote host development, metabolism, lifespan, growth performance, production, and fecundity. The gut microbiota can affect key aspects of host physiology and play important roles in host fitness. However, details concerning the gut bacteria of snails remain poorly understood.

Gastropoda has the largest number of species among the classes in the phylum Mollusca, including more than 75,000 species of gastropods [13], some of which are intermediate hosts of parasites [14,15]. In recent years, the understanding of gut bacterial composition in gastropods has improved. For instance, *Achatina fulica*, a terrestrial gastropod, harbors diverse gut microbes, including the dominant bacteria *Lactococcus lactis* and *Kurthia gibsonii*, which are present in all regions of its gastrointestinal tract [16,17]. *Pomacea canaliculata* exhibits dominant gut bacteria, namely *Leuconostoc* (45.68%) and *Lactococcus* (20.64%), which are associated with the developmental stage and sex of the snail [18]. Other species, such as *Biomphalaria glabrata* [19], *Planorbella trivolvis* [20], *Oreohelix strigosa* [21], and *Theodoxus fluviatilis* [22], have provided a general understanding of the characteristics of gut bacterial diversity and communities of gastropods. Furthermore, an increasing number of studies have focused on the snail gut microbiota, demonstrating its strong association with snail fitness [14,15]. However, our knowledge of the assembly processes of the gut microbiota is still limited.

*Biomphalaria straminea*, the intermediate host of *Schistosoma mansoni*, has invaded China, posing a high potential risk of *S*. *mansoni* transmission in Southern China [23]. Our previous study used 16S rRNA gene sequencing and metagenomics to reveal the gut microbiota composition and diversity of *B*. *straminea* [13,24]. We suggested a strong association between the gut microbiota and snail fitness. Additionally, regarding to microbial community assembly, two fundamental processes are often highlighted: stochastic and deterministic processes [25]. Stochastic processes, as described by neutral theory, involve random, non-selective events such as birth, death, immigration, and ecological drift [26]. While deterministic processes are driven by specific, non-random factors that shape community composition in a more predictable manner [26]. Both stochastic and deterministic processes play crucial roles in the assembly of microbial communities, including the fish gut microbiota [27–30]. However, whether the balance between stochastic and deterministic processes in the snail gut microbiota is associated with snail fitness remains unclear.

In this study, we aimed to analyze the processes that determine the gut microbiota assembly of *B*. *straminea* and determine the relationship between stochastic and deterministic processes of the snail gut microbial composition and snail adaptation.

## Methods

### Snail husbandry

The cultured strain of *B*. *straminea* snails in the laboratory has been maintained for over 10 years. Wild-caught *B*. *straminea* snails collected from Shenzhen (22°33’27"N, 114°8’40"E) were transferred to the Department of Parasitology at Sun Yat-sen University for further study. The snails were fed sterile fish food, which included ingredients of animal and plant origin, such as fish meal, wheat flour, corn protein, yeast and spirulina flour, and were reared in a controlled environment. The sterile food was prepared through autoclave sterilization. The snails were housed in an aquatic tank filled with dechlorinated water, which was renewed every 2 days, and kept at a temperature of 25 to 27°C with 80% relative humidity (RH) and a 12:12 h light-dark (L:D) cycle.

### Sample collection

For different habitats under different laboratory conditions, Habitat 1 used dechlorinated filtered water, while Habitat 2 used tap water that was allowed to aerate for at least 24 h before being used to replenish the snails with fresh dechlorinated water. At least 10 samples from snails (7 to 8 weeks old) were collected from each habitat. Field surveys were conducted in Shenzhen and Hong Kong cities. The wild snails were transferred to the laboratory and dissected immediately after being caught. Before being dissected, each snail was surface-sterilized in sterile water for 30 seconds and washed three times in different sterile dishes. It was then rinsed in sterile phosphate-buffered saline (PBS) for 15 seconds. Subsequently, the snail shell was removed, and the body was rinsed twice with sterile PBS for 15 seconds each. The body was then dissected in a sterile dish, and the gut was isolated. The gut samples for sequencing were directly stored at -80°C until further processing.

### DNA extraction

Each sequenced sample was made up of either one gut from a single snail or guts from two snails combined into one sample. The collected gut sample in a tube was supplemented with 15 μL of sterile ddH2O and homogenized using an electric homogenizer on ice, following the procedure outlined by Lin and colleagues [31]. Total DNA extraction from the *B*. *straminea* gut sample was performed according to the manufacturer’s protocol using the HiPure Bacterial DNA Kit (Magen, China). DNA quantity and quality were assessed using a Nanodrop (Thermo, USA). Total DNA was suspended in 30 μL of nuclease-free buffer (Magen, China) and stored at -80°C until further analysis.

### Amplification, library preparation and sequencing

The hypervariable regions (V3-V4) of the 16S rRNA gene were amplified using universal-specific primers. The following 341F/806R primer sets were used for polymerase chain reaction (PCR): forward 5’-CCTAYGGGRBGCASCAG-3’ and reverse 5’-GGACTACNNGGGTATCTAAT-3’. All PCR reactions were carried out with 15 μL of Phusion High-Fidelity PCR Master Mix (New England Biolabs). The PCR conditions were set as follows: initial denaturation at 98°C for 1 min, followed by 30 cycles of 98°C for 10 s, 50°C for 30 s, and 72°C for 30 s, and final extension at 72°C for 5 min. To test for the presence of possible contaminants, we showed the microbiome control (kitome control) as the control group. One PCR was performed for each sample. PCR products were visualized on 2% agarose gel electrophoresis and purified using the Qiagen gel extraction kit (Qiagen, Germany) according to the manufacturer’s instructions.

Sequencing libraries for 16S rRNA gene sequencing were generated using the TruSeq DNA PCR-Free Sample Preparation kit (Illumina, USA). The library quality was assessed on the Qubit 2.0 Fluorometer (Thermo, USA) and the Agilent Bioanalyzer 2100 system (Agilent, USA). Thereafter, the 16S rRNA gene (V3-V4 region) library was sequenced on an Illumina PE250 platform constructed by the Novogene company (Novogene, China), and paired-end reads were generated.

### Microbiome data analysis

The bacterial data were obtained based on 16S rRNA gene sequencing. We obtained sequences after sequence assembly, with paired-end reads merged using FLASH (V1.2.7) [32]. To obtain high-quality clean tags, raw tags were quality-filtered using QIIME (v. 1.9.1) [33,34]. QIIME (v. 1.9.1) was used to cluster reads into OTUs. Sequence analysis was carried out by Uparse software (v. 7.0.1001) [35]. Sequences with 97% or greater similarity were assigned to the same OTUs. Rarefaction curve analysis and niche width were performed using R software (v. 2.15.3). Each representative sequence was identified and annotated with taxonomic information using the Silva Database (v. 138.1) [36]. Because of the high similarity, rare taxa with an abundance of 0.001% were excluded.

To analyze alpha diversity, we rarified the OTU table and calculated metrics: observed species and Shannon index [37]. Principal coordinate analysis (PCoA) graphs (based on the Bray–Curtis distance) as measures of beta diversity were performed in R using the WGCNA package, stat package, and ggplot2 package [38]. A Raup-Crick (RC) comparison was conducted using a normalized stochasticity ratio in R [39]. Venn diagram and transmission analysis were performed in R statistical software using the vegan packages. Permanovas were conducted using the Adonis function in the Vegan package in R to analyze the significance of differences in bacterial community structure between groups. To further analyze the determined factors in gut microbiota assembly, the balance of stochastic and deterministic processes was analyzed using the neutral community model (NCM) [40]. The neutral community model (NCM) was performed as described in a previous study [41]. The NCM assumes that all species in a metacommunity are ecologically equivalent and community dynamics are driven by stochastic processes, including random birth, death, and dispersal. The model predicts that more abundant taxa are widely dispersed, while rare taxa are more likely to be lost due to ecological drift, with key parameters including the metacommunity size (N) and immigration rate (*m*). Deviations from the NCM predictions were analyzed by partitioning OTUs into groups based on their occurrence relative to the model’s 95% confidence intervals and comparing their diversity, composition, and estimated migration rates. To further evaluate the relative significance of stochasticity and determinism in the snail gut bacterial assembly, a two-step procedure was employed, considering the βNTI and RC_bray_ values [42]. This assessment encompassed various ecological processes, categorized into stochastic (undominated processes, homogenizing dispersal, and dispersal limitation) and deterministic (homogeneous selection and variable selection), with βNTI and RC_bray_ values computed using the R package iCAMP (v1.3.4). [42]. All pairwise comparisons of each pair of groups (Habitat 1 versus Habitat 2, Adaptive vs. Non-adaptive) were analyzed by using the Wilcoxon test or *T*-test for range adjustment. Statistical significance was defined at *P* <0.05.

## Results

### Diversity and composition of the gut microbiota in *Biomphalaria straminea*

**Fig 1:**
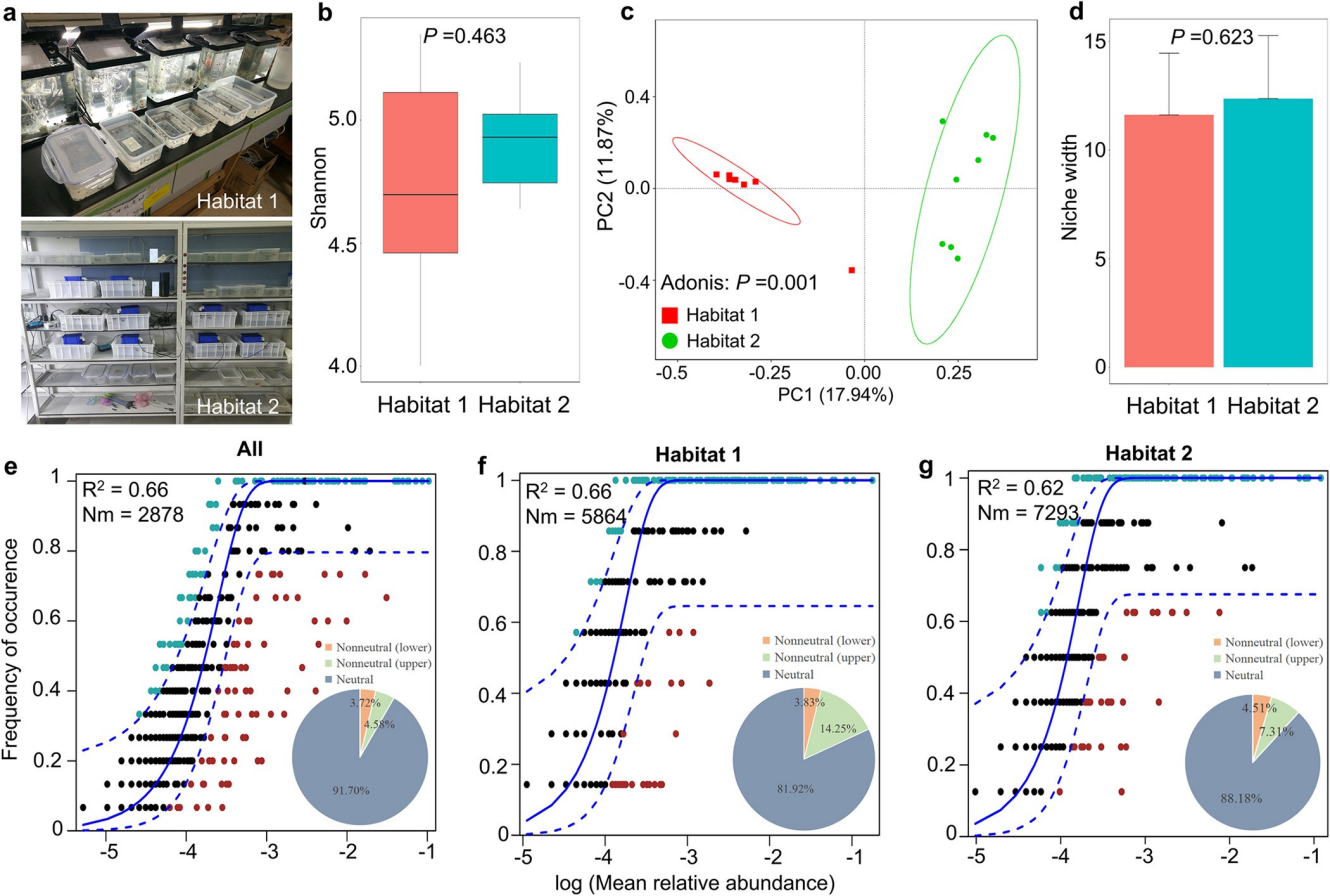
Overview of the gut microbial assembly of *B*. *straminea* (snail sample size: n = 7 from habitat 1; n = 8 from habitat 2) and fit of neutral models for snails from different habitats. **a** Showing different habitats under different laboratory maintenance conditions. **b** Shannon index. **c** Principal coordinates analysis (PCoA). **d** Niche width. **e-g** Fit of the neutral community model (NCM) of gut bacterial community assembly in *B*. *straminea*. OTUs that occur more (blackish green) or less (red) frequently than predicted (blue) by the NCM were shown in different colors. The solid blue lines indicate the best fit for the NCM. The dashed lines indicate the 95% confidence interval (CI) around the neutral model. R^2^: the fit to the model. Nm: the metacommunity size times immigration. All: including snails from both habitat 1 and habitat 2. In the pie charts, gray represents the proportion of neutral predictions in the NCM, orange represents the proportion of lower nonneutral predictions, and green represents the proportion of upper nonneutral predictions. Neutral predictions indicate an association with stochastic processes, while nonneutral predictions are associated with deterministic processes. *P* <0.05 is considered statistically significant.

*B*. *straminea* snail gut samples for 16S rRNA gene sequencing were collected from different habitats and housed under different laboratory conditions (**Fig 1A**). After being processed and filtered, the sequences were classified into operational taxonomic units (OTUs) with 97% minimum identity. The snail gut bacterial rarefaction curves are shown (**S1 Fig a**). While the diversity of gut microbiota does not significantly differ between snails from different habitats (*P* > 0.05), the composition of gut microbial communities shows significant variation (*P* <0.05) (**Fig 1B and 1C**). The dominant gut microbe in snails, regardless of their habitat, is from the phylum Proteobacteria (Habitat 1: 51.31%; Habitat 2: 66.58%), followed by Bacteroidota (Habitat 1: 35.90%; Habitat 2: 27.31%) (**S1 Fig b**). At the genus level, gut microbes such as *Aeromonas* (29.83%), *Cloacibacterium* (12.13%), *Flavobacterium* (9.91%), and *Acinetobacter* (7.90%) show a higher abundance in snails from habitat 2, while snails from habitat 1 harbor a higher abundance of *Chryseobacterium* (17.87%), *Pseudomonas* (10.57%), and *Cetobacterium* (5.84%). Additionally, the Venn diagram analysis of snail gut microbiomes shows the number of shared bacterial OTUs (n = 358) in both groups (**S2 Fig**). These findings indicate that while snails from different habitats share some dominant gut microbes, the abundance of these bacteria varies.

### Stochastic processes govern gut bacterial community assembly in *Biomphalaria straminea*

**Fig 2:**
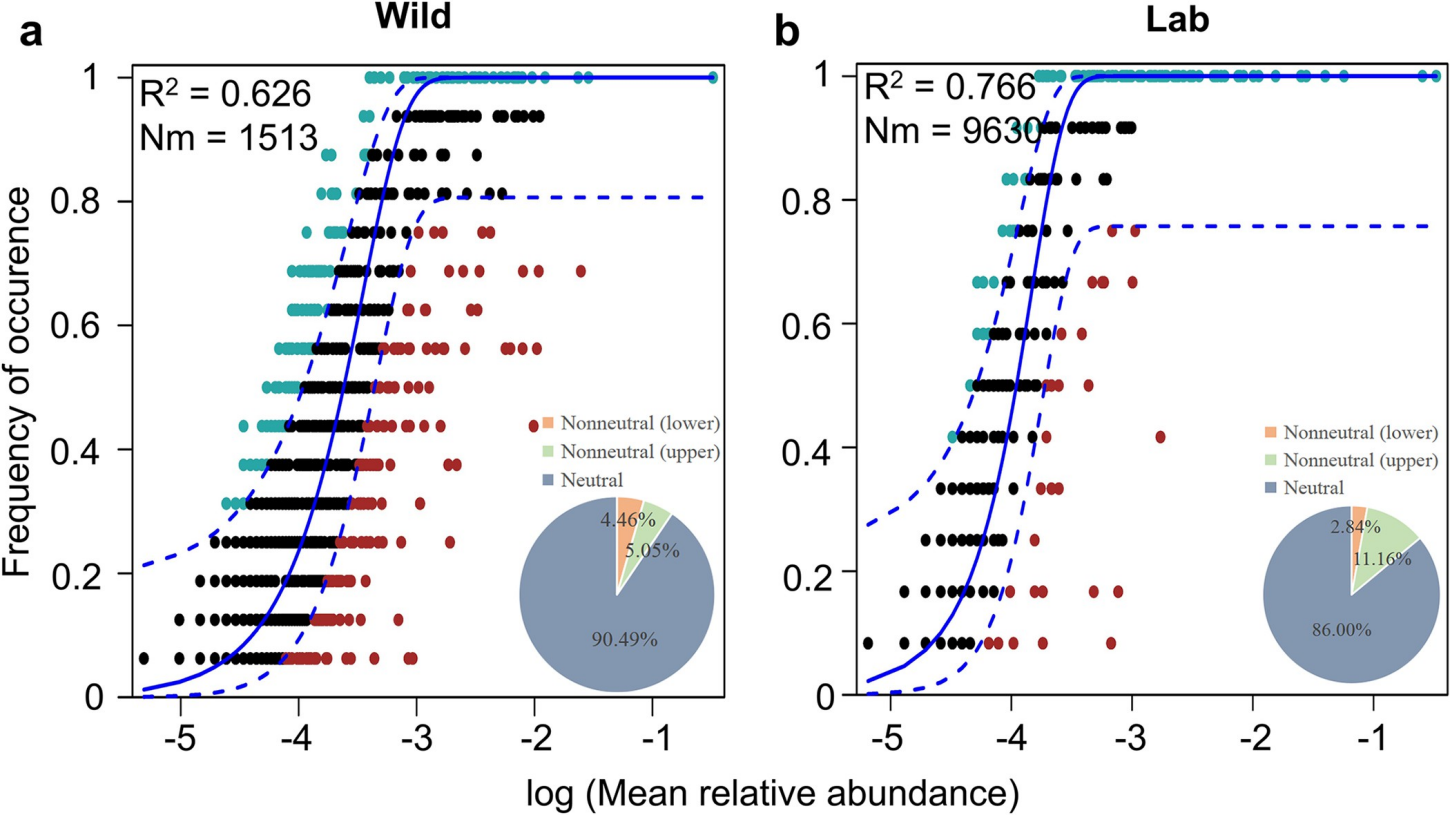
Fit of NCM of snail gut bacterial community assembly for wild (n = 16) and laboratory (n = 12) snail samples. **a** Wild snail. **b** Laboratory snails. OTUs that occur more (blackish green) or less (red) frequently than predicted (blue) by the NCM were shown in different colors. The solid blue lines indicate the best fit for the NCM. The dashed lines indicate the 95% confidence interval (CI) around the neutral model. R^2^: the fit to the model. Nm: the metacommunity size times immigration. In the pie charts, gray represents the proportion of neutral predictions in the NCM, orange represents the proportion of lower nonneutral predictions, and green represents the proportion of upper nonneutral predictions. Neutral predictions indicate an association with stochastic processes, while nonneutral predictions are associated with deterministic processes.

We found that a large fraction of the relationship between the occurrence frequency of OTUs and their relative abundance could be predicted by the NCM. Our neutral community model observed 91.70% of the community variance for the entire dataset, with 81.92% and 88.18% of the variance explained for habitat 1 and habitat 2, respectively (**Fig 1E–1F**). In addition, we further analyzed the gut microbial assembly of *B*. *straminea* snails from wild and laboratory habitats and found that the assembly process of the snail gut microbiota is more stochastic than deterministic (**Fig 2**).

### The balance between stochastic and deterministic processes in gut microbial composition is associated with snail adaptation

**Fig 3:**
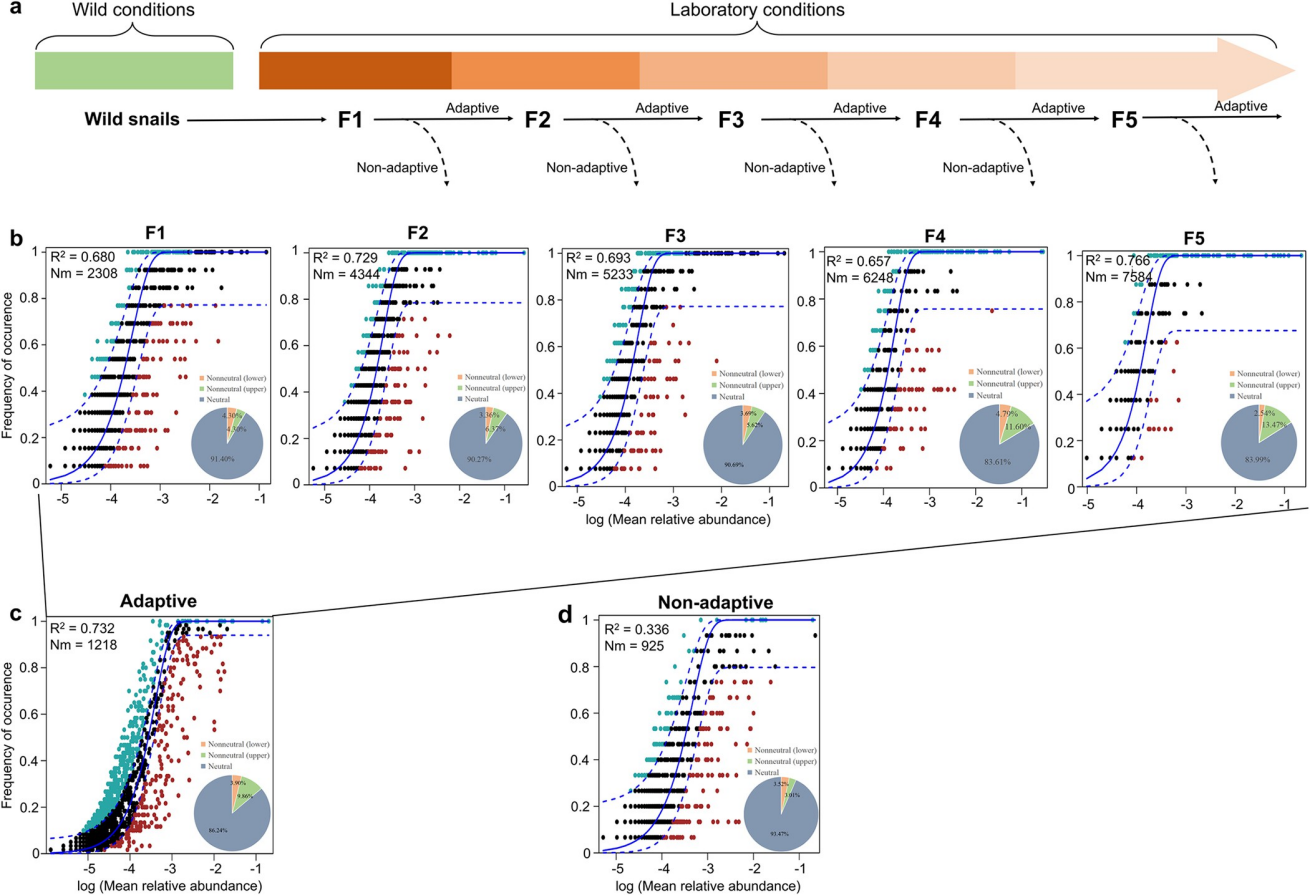
Fit of NCM of snail gut bacterial community assembly across generations during snail adaptation to a new environment. **a** The study design and sample collection from different generations were conducted, with some samples referenced from a previous study [13]. **b** Snail populations from different generations (snail sample size: F1, n = 13; F2, n = 14; F3, n = 13; F4, n = 12; F5, n = 8). **c** Adaptive snails (snail sample size: n = 60). **d** Non-adaptive snails (snail sample size: n = 15). OTUs that occur more (blackish green) or less (red) frequently than predicted (blue) by the NCM were shown in different colors. The solid blue lines indicate the best fit for the NCM. The dashed lines indicate the 95% confidence interval (CI) around the neutral model. R^2^: the fit to the model. Nm: the metacommunity size times immigration.

We further analyzed the gut microbiota of *B*. *straminea* snails across the F1 to F5 generations during their offspring’s adaptation to a new environment (**Fig 3A**). During the process of offspring adapting to a new environment, successful individuals (adaptive snails) can lay eggs and reproduce, while unsuccessful (non-adaptive snails) may die or fail to reproduce (**Fig 3A**). Using the NCM, we identified a substantial portion of the relationship between the occurrence frequency of OTUs and their relative abundance, explaining 91.40%, 90.27%, 90.69%, 83.61%, and 83.99% of the community variance for the F1, F2, F3, F4, and F5 generations, respectively (**Fig 3B**). Furthermore, for these adaptive snails, the neutral community model (NCM) provided a strong fit for the gut bacterial OTUs, with 86.24% of the OTUs falling within the 95% confidence interval (CI), indicating that stochastic processes predominantly shape the gut microbiota (**Fig 3C**). The impact of neutral processes on the gut microbiota assembly in non-adaptive snails is similar to that observed in adaptive individuals. The fit of gut bacterial OTUs to the NCM is more robust, with only 6.53% of the OTUs falling outside the 95% CI (**Fig 3D**). These findings suggest that during the period when the snails were placed in a new environment, stochastic processes are more crucial than deterministic processes in shaping the snail gut microbiota.

### Mechanisms for driving the gut bacterial community assembly in *Biomphalaria straminea* using null model analysis

**Fig 4:**
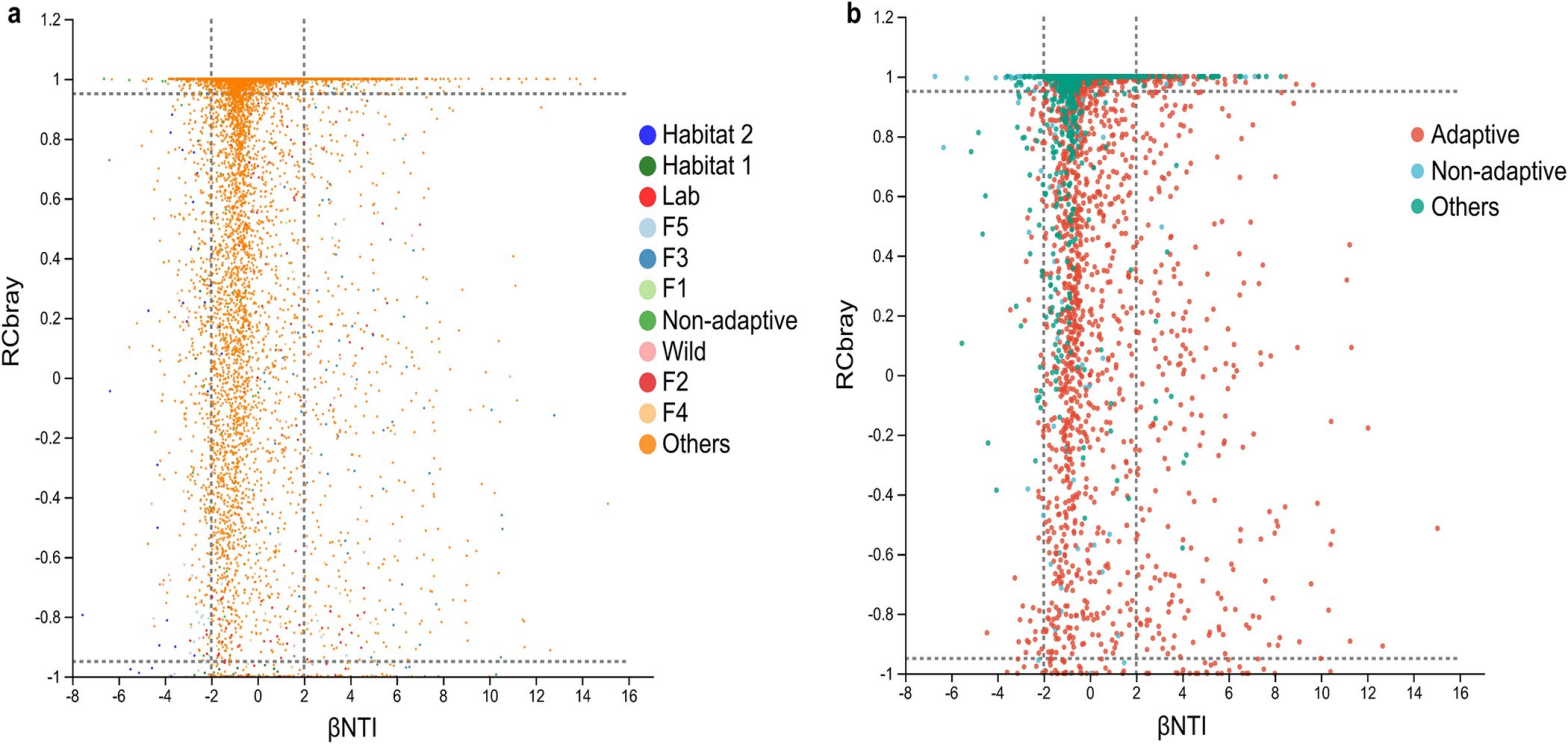
Graphs of βNTI/RC_bray_ for snail gut bacterial community structure analysis. **a** For all samples (n = 118). **b** For adaptive (n = 60) and non-adaptive (n = 15) samples. The horizontal dashed lines represent the values -2 and 2. The vertical dashed lines represent the values -0.95 and 0.95. When βNTI > 2, it indicates heterogeneous selection, while βNTI < -2 indicates homogeneous selection. When |βNTI| ≤ 2, RC_bray_ > 0.95 suggests dispersal limitation, RC_bray_ < -0.95 suggests homogenizing dispersal, and |RC_bray_| ≤ 0.95 indicates undominated. In the legend, different groups represent pairwise comparisons within the same group, while ’Others’ refers to comparisons between samples from different groups. RC_bray_: Bray–Curtis-based Raup–Crick Index. βNTI: β-nearest taxon index.

**Fig 5:**
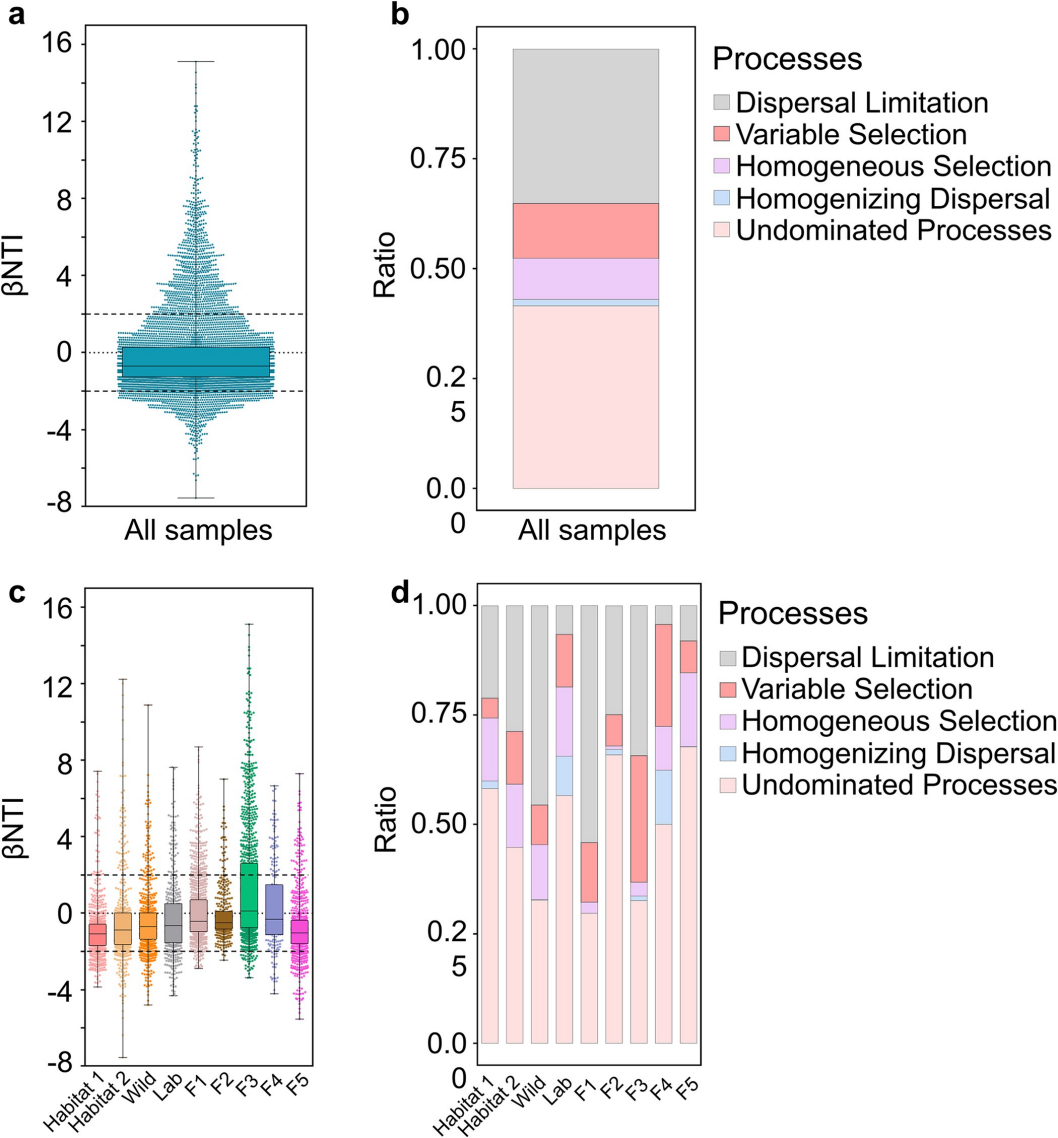
Mechanisms of snail gut bacterial community assembly evaluated using null model analysis. **a** Contributions of stochastic processes (|βNTI | <2) and deterministic processes (|βNTI |  ≥2) on the gut bacterial community assembly of all snail samples (n = 118). The horizontal dashed lines represent the values -2 and 2. **b** The ratio of different ecological processes (dispersal limitation: |βNTI| < 2 and RC_bray_ > 0.95, variable selection: βNTI < −2, homogeneous selection: βNTI > 2, homogenizing dispersal: |βNTI| < 2 and RC_bray_ <−0.95, and undominated processes: |βNTI|  < 2 and |RC_bray_|  < 0.95) in all snail sample groups. **c** Contributions of stochastic processes and deterministic on gut bacterial community assembly in different groups. The horizontal dashed lines represent the values -2 and 2. **d** The ratio of different ecological processes in different groups. RC_bray_: Bray–Curtis-based Raup–Crick Index. βNTI: β-nearest taxon index.

To further quantify the relative impact of stochastic and deterministic forces in shaping the freshwater snail gut bacterial community assembly, the null model was used. Based on the beta nearest taxon index (βNTI) and Bray-Curtis-based Raup-Click index (RC_bray_), the majority of the gut bacterial community structure for snail samples falls between 2 and -2 (**Fig 4A and 4B and Fig 5A**). For the overall snail samples, microbial assembly was more strongly influenced by stochastic processes (dispersal limitation: 35.09%, homogenizing dispersal: 1.47%, and undominated processes: 41.55%) than by deterministic processes (variable selection: 12.51% and homogeneous selection: 9.39%) (**Fig 5B**).

### Mechanisms driving gut bacterial community assembly during snail adaptation to a new environment

Additionally, stochastic processes (dispersal limitation, homogenizing dispersal, and undominated processes) are more important than deterministic processes (variable selection and homogeneous selection) in gut bacterial communities in the F1, F2, F3, F4, and F5 generations, respectively (**Fig 5C and 5D**). Undominated processes (F1: 29.68%, F2: 65.90%, F3: 32.61%, F4: 50.00%, and F5: 67.69%) and dispersal limitation (F1: 54.12%, F2: 24.87%, F3: 34.26%, F4: 4.29%, and F5: 8.08%) were the two primary factors governing the assembly of bacterial communities in snail offspring across the F1 to F5 generations.

Interestingly, there is an increasing trend of proportions of homogeneous selection across F1-F5 generations (F1: 2.53%, F2: 0.77%, F3: 3.14%, F4: 10.00%, and F5: 16.79%) and variable selection (F1: 13.67%, F2: 7.18%, F3: 28.95%, F4: 23.33%, and F5: 7.31%), alongside a decreasing trend in dispersal limitation (F1: 54.12%, F2: 24.87%, F3: 34.26%, F4: 4.29%, and F5: 8.08%) in the gut bacterial communities of snail offspring across the F1-F5 generations. These trends may be associated with the adaptation process of snail offspring to a new environment (**Fig 5D**).

**Fig 6:**
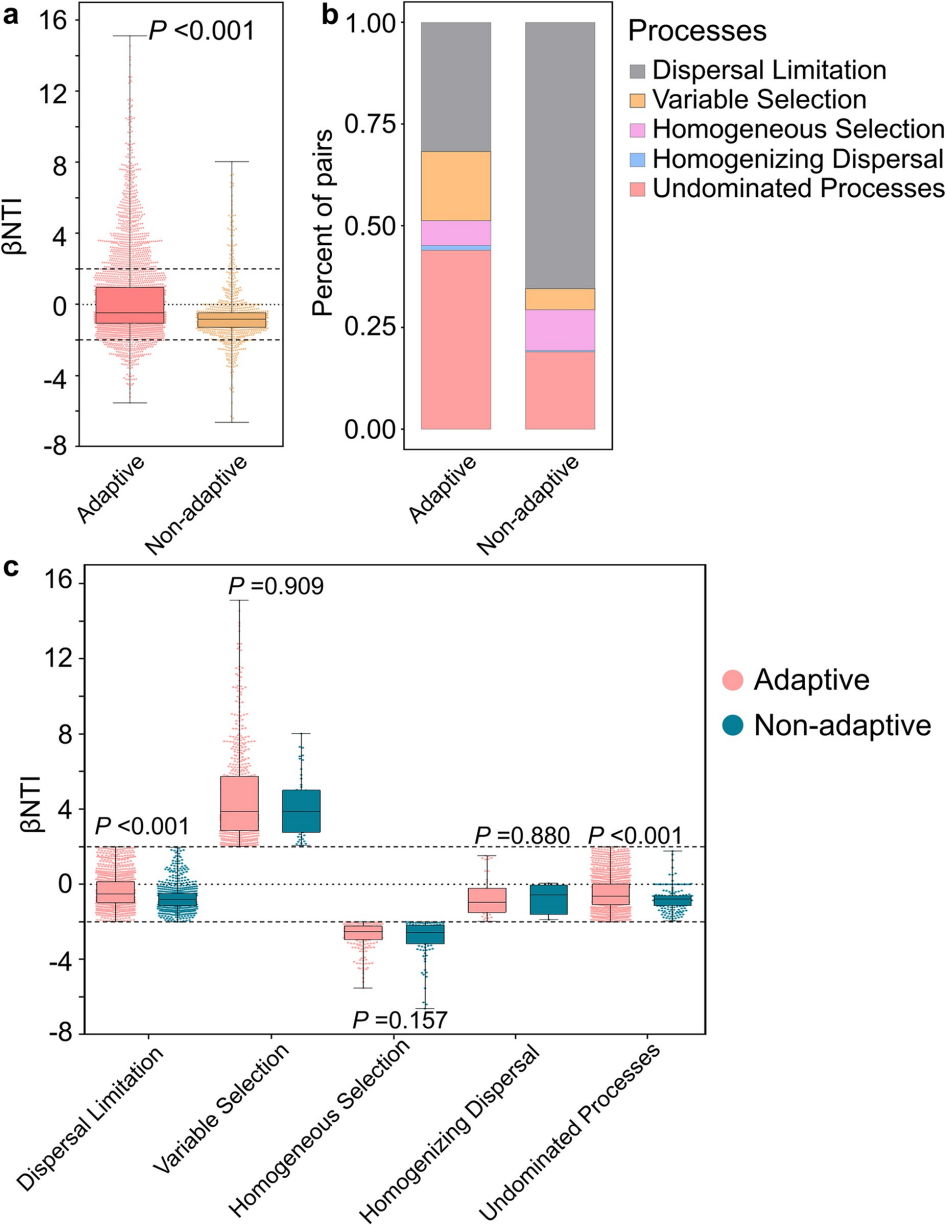
Mechanisms of gut bacterial community assembly in adaptive (n = 60) and non-adaptive (n = 15) snails evaluated using null model analysis. **a** Contributions of stochastic processes (|βNTI |  <2) and deterministic processes (|βNTI |  ≥2) on gut bacterial community assembly of different snail samples. The vertical dashed lines represent the values -2 and 2. When βNTI > 2, it indicates heterogeneous selection, while βNTI < -2 indicates homogeneous selection. When |βNTI| ≤ 2, RC_bray_ > 0.95 suggests dispersal limitation, RC_bray_ < -0.95 suggests homogenizing dispersal, and |RC_bray_| ≤ 0.95 indicates undominated. **b** The ratio of different ecological processes (dispersal limitation: |βNTI| < 2 and RC_bray_  > 0.95, variable selection: βNTI  < −2, homogeneous selection: βNTI  > 2, homogenizing dispersal: |βNTI| < 2 and RC_bray_  <−0.95, and undominated processes: |βNTI|  < 2 and |RC_bray_|  < 0.95) in different groups. **c** Differences in contributions of different ecological processes on gut bacterial community assembly between different groups. The vertical dashed lines represent the values -2 and 2. RC_bray_: Bray–Curtis-based Raup–Crick Index. βNTI: β-nearest taxon index. *P* <0.05 is considered statistically significant.

During the adaptation process of offspring to a new environment, the gut bacterial communities of adaptive snails exhibit significantly higher βNTI values compared to those of non-adaptive snails (**Fig 4B and 6A**). Additionally, compared to non-adaptive snails, adaptive snails exhibited lower proportions of undominated processes (43.98% versus 18.99%) and variable selection (17.02% vs. 5.22%) in their gut bacterial communities. Conversely, adaptive snails showed higher proportions of dispersal limitation (31.71% vs. 65.43%) and homogeneous selection (6.06% vs. 9.97%). These differences may have significantly influenced the gut bacterial community structures during snail adaptation (**Fig 6B and 6C**). A significant difference in the proportions of dispersal limitation and undominated processes is evident between the two types of snail samples (**Fig 6C**).

## Discussion

The gut microbiota, shaped and influenced by multiple factors [14,21,27,43–45], play a crucial role in host development and biology. However, understanding the mechanisms of community assembly remains a critical yet limited topic in freshwater snail microbial ecology. Here, we focused on the gut bacterial compositions of *B*. *straminea* and investigated their assembly mechanisms. We demonstrated that habitats from different rearing conditions significantly affect the snail gut microbial community, resulting in varying relative abundances. Importantly, this study revealed that stochastic processes dominate over deterministic processes in shaping snail gut microbiota composition, and the balance between stochastic and deterministic processes in gut microbial composition is associated with snail fitness during snail adaptation to a new environment. To our knowledge, this is the first evidence showing that stochastic and deterministic processes can shape the gut microbiota composition in *S*. *mansoni*-transmitting snails. Findings from this study will enhance our understanding of the snail gut microbiota and its potential mechanisms underlying the factors that determine the gut microbiome of snail intermediate hosts of schistosomiasis.

Habitat differences affect the composition of the snail gut microbiomes but do not influence the assembly mechanism. Our study shows that the dominant gut microbes, Proteobacteria and Bacteroidota, are consistently present in *B*. *straminea* across different habitats. But many animals, like mammals and fish, harbor the phyla Firmicutes and Proteobacteria, or Firmicutes and Bacteroidetes, as dominant bacteria in their guts [8,46–52]. Previous studies have revealed that *Prevotella*, *Bacteroides*, and *Ruminococcaceae* are the three dominant gut taxa in mammals [53–57], which are different from the dominant bacterial taxa in *B*. *straminea* snails, including *Aeromonas*, *Cloacibacterium*, *Flavobacterium*, *Acinetobacter*, *Chryseobacterium*, and *Pseudomonas*. These studies indicated that the potential differences in gut microbiota were associated with a range of complex factors, such as habitats and species.

Furthermore, ecological succession and the balance between deterministic and stochastic processes are two major themes in microbial ecology [26]. Deterministic processes are associated with ecological selection, including abiotic and biotic factors that determine the presence, absence, and relative abundances of species [25,26]. Stochastic processes are not the consequence of environmentally determined fitness but involve probabilistic dispersal and random changes in species’ relative abundances [26]. Previous studies have revealed the mechanisms by which the river microeukaryotic metacommunity across different hydrographic regimes is primarily determined by stochastic processes, with 89.9%, 88.5%, and 89.6% of the community variation explained by the neutral community model during the wet, dry, and both seasons, respectively [41,58]. Stochastic processes are important in shaping the gut microbiota of animals like shrimps and *Helicoverpa armigera* [29]. In freshwater snails, the authors have demonstrated that deterministic assembly constrains the diversity of the gut microbiota of *P*. *canaliculata* [27]. However, our study reveals that stochastic processes predominantly influence the gut microbiota communities of snails across different habitats and generations. This also contrasts with findings that deterministic processes dominate gut bacterial community assembly in fish and *Moschus chrysogaster* [28,59]. Stochastic processes are driven by random, unpredictable events like birth, death, and dispersal, while deterministic processes are influenced by specific, non-random factors that shape community composition in a more predictable way. We speculate that the differences in findings may be primarily related to species-specific factors. Additionally, our study focuses more on stable habitats and different generations, whereas previous studies have considered factors like geography. This may explain why stochastic and deterministic processes shape the host gut microbiota differently. Furthermore, previous research has also shown that the gut microbiota of invasive and native snails differs in terms of the dominance of deterministic and stochastic processes [27]. Overall, the interplay between stochastic and deterministic processes shapes the gut microbial composition of animals.

Niche processes indicate that microbial communities are associated with abiotic factors [25,26]. Recent studies have demonstrated that environmental habitats can shape the host gut microbiota and serve as important sources of animal gut microbiota [52,60]. However, these studies on the establishment of gut microbiota were mainly related to vertebrates. In a recent study, the authors demonstrated that deterministic processes constrain the diversity of the gut microbiota in the freshwater snail, *P*. *canaliculata* [27]. In contrast, our study reveals the close associations between stable habitats and the assembly of the snail gut microbiota, showing that stochastic processes govern gut bacterial community assembly in the *S*. *mansoni*-transmitting snail, *B*. *straminea*. Furthermore, while habitat changes significantly affect the gut microbiota composition of snails, they do not strongly alter the assembly processes. Even when the habitat changes from the wild to laboratory conditions, stochastic processes remain the major factor shaping the gut microbiota in *B*. *straminea*, rather than deterministic processes.

Previous studies have reported that freshwater snails, such as intermediate hosts of *Schistosoma mansoni* like *B*. *straminea* and *B*. *glabrata*, originating from the southeastern part of South America, have spread to various water habitats in countries or regions such as Brazil, Paraguay, Argentina, Uruguay, Colombia, Grenada, Guadeloupe, and Puerto Rico [23,61,62]. This implies that these freshwater snails have diverse habitat sources. Here, during the process of snail invasion and adaptation to a new environment, stochastic processes play a more critical role than deterministic processes in shaping the snail gut microbiota. Additionally, the animal gut microbiota is associated with host fitness [13,63]. This study also shows that the balance between stochastic and deterministic processes in snail gut microbial assembly is associated with host fitness during snail adaptation to a new environment. Furthermore, the proportions of dispersal limitation and undominated processes are associated with the adaptive and non-adaptive snail samples. Therefore, considering the impact of shaping factors on snail gut bacterial assembly processes, particularly dispersal limitation and undominated processes, habitat changes may provide insights into understanding the gut microbiota of intermediate hosts. This understanding can aid in designing effective strategies for schistosome research and the elimination of schistosomiasis.

## Conclusions

In summary, we analyzed the gut bacterial compositions of the freshwater snail *B*. *straminea* and revealed potential bacterial assembly mechanisms. We showed that habitats significantly affect the snail gut microbial community. We also indicated that stochastic processes dominate over deterministic processes in shaping the gut microbial assembly of snails from different habitats using NCM. Our study presented a comprehensive view of the dynamic changes in snail gut microbiota during snail adaptation to a new environment and provided further insights into the mechanism of driving the gut bacterial community assembly in *B*. *straminea* using null model analysis. Additionally, this study shows an increasing trend in the proportions of deterministic processes (based on homogeneous selection and variable selection) in the gut bacterial communities of snail offspring, indicating that the balance between stochastic and deterministic processes in the gut microbial composition is associated with snail adaptation. The significant difference in the proportions of dispersal limitation and undominated processes is associated with adaptive and non-adaptive snails. Our study enhances our understanding of the snail gut microbiota and advances our knowledge of bacterial assembly mechanisms, offering insights for the development of control strategies against snails and snail-borne diseases.

## Supporting information

S1 Fig Overview of gut microbiomes of *Biomphalaria straminea* from different laboratory habitats. **a** Rarefaction analysis of observed OTUs from snail bacterial samples. **b** At the phylum level. **c** At the genus level.
(TIF)

S2 Fig Venn diagram analysis of gut microbiomes of *Biomphalaria straminea* from different laboratory habitats. (TIF)
