# Supplementary figures and images for: Stochastic processes govern gut bacterial community assembly in a Schistosoma mansoni-transmitting snail, Biomphalaria straminea
Source: PLoS Negl Trop Dis. 2025 Feb 5;19(2):e0012828. doi: 10.1371/journal.pntd.0012828 (PMC11798439; doi:10.1371/journal.pntd.0012828)

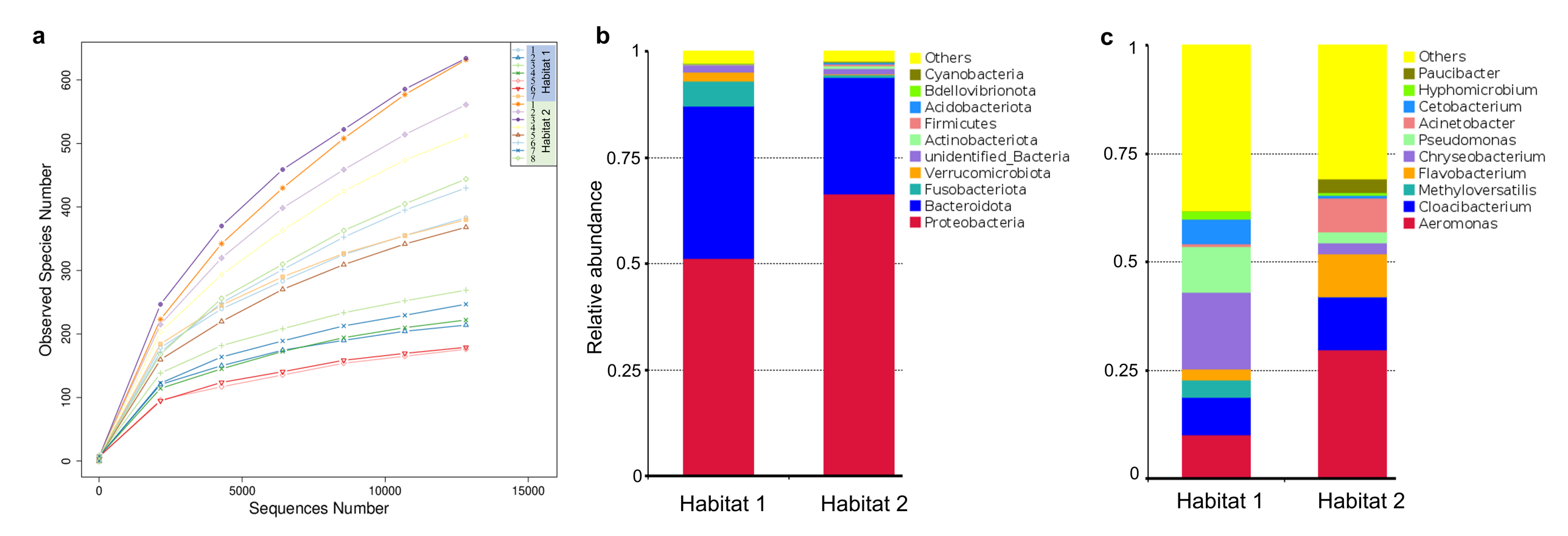

Supplement: S1 Fig — a Rarefaction analysis of observed OTUs from snail bacterial samples. b At the phylum level. c At the genus level. (TIF) [file pntd.0012828.s001.tif]

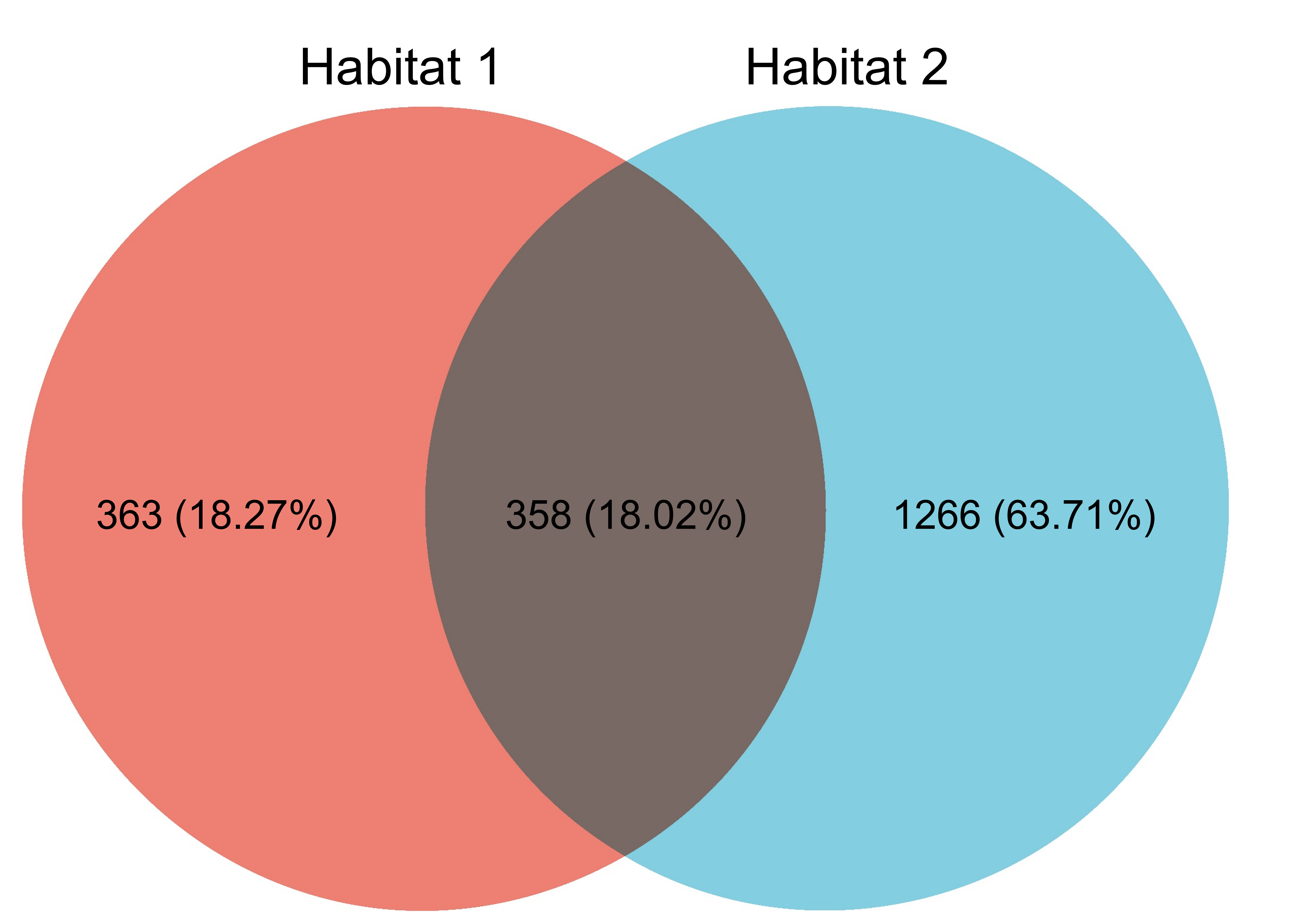

Supplement: S2 Fig — (TIF) [file pntd.0012828.s002.tif]
